# Pregnancy Outcomes of Women Veterans with Autoimmune Disease

**DOI:** 10.1089/whr.2024.0078

**Published:** 2024-09-06

**Authors:** Catherine A. Sims, Dahima Cintron, Kate Wallace, Aimee Kroll-Desrosiers, Ankoor Shah, Jennifer M. Gierisch, Karen M. Goldstein, Kristin Mattocks

**Affiliations:** ^1^ Department of Medicine, Durham Veterans Affairs Healthcare System, Durham, North Carolina, USA.; ^2^ Division of Rheumatology, Department of Medicine, Duke University, Durham, North Carolina, USA.; ^3^ Department of Medicine and Pediatrics, Division of Rheumatology, Duke University, Durham, North Carolina, USA.; ^4^ VA Central Western Massachusetts VA Healthcare System, Leeds, Massachusetts, USA.; ^5^ Department of Population and Quantitative Health Sciences, University of Massachusetts Chan Medical School, Worcester, Massachusetts, USA.; ^6^ Division of Internal Medicine, Duke University School of Medicine, Durham, North Carolina, USA.; ^7^ Department of Population Health Sciences, Duke University School of Medicine, Durham, North Carolina, USA.

**Keywords:** pregnancy, autoimmune disease, Veterans, reproductive health

## Abstract

**Background/Objective:**

Women Veterans (WV) are exposed to unique risk factors for the development of autoimmune diseases (AID), which can increase risk of pregnancy complications. To characterize pregnancy outcomes in this population, our team performed a descriptive case series.

**Methods:**

To identify WV with AID from the Center for Maternal and Infant Outcomes Research in Translation dataset, medical records were screened using diagnostic codes and medications. A protocolized chart review and extraction was performed.

**Results:**

Twenty-five WV with AID were identified. The most frequently reported AID were inflammatory bowel disease (*n* = 4), psoriasis (*n* = 4), and undifferentiated connective tissue disease (*n* = 4). Forty-four percent of WV with AID experienced pregnancy complications, 32% utilized VA subspecialty care for AID management, and 40% did not seek health care at the VA during their pregnancy.

**Conclusions:**

Identified pregnancies had a high frequency of complications with more than one in three Veterans lost to VA follow-up during pregnancy.

## Background

Women with complex and chronic conditions, including autoimmune diseases (AID), are at higher risk of poor pregnancy outcomes, including preeclampsia and preterm delivery, compared with women without these diseases.^1^ The risk of these poor pregnancy outcomes is unique for each type of AID. However, characteristics in all AID that increase the risk of adverse pregnancy outcomes include increased disease activity, presence of specific autoantibodies, and history of co-existing hypertension, which can be a comorbidity or result of the AID and/or its therapies.^1^ Moreover, AID impact medical costs, long-term health, quality of life, chronic pain burden, daily functioning, mental health, and reproductive health across the entire lifespan.^2–8^

Sex-based differences in the incidence and complications of AID have important implications for women Veterans (WV). WV are up to five times more likely than male Veterans to be diagnosed with AID.^9^ In 2015, more than 40,000 WV receiving care in the VA health care system carried an AID diagnosis.^9^ The unique experiences of Veterans, such as deployment and its associated activities, increase their risk of the development of AID.^10,11^ For example, the Millennium Cohort Study cited post-traumatic stress disorder (PTSD) and infectious gastroenteritis as risk factors for diagnosis of AID.^12^ Additionally, more than 300,000 WV have participated in military operations involving burn pit exposure, which is associated with a threefold increase in the diagnosis of an AID and a near twofold increase in the presence of autoantibodies, which can serve as a risk factor for the development of AID.^13–16^

WV are more likely to experience additional adverse events such as military sexual trauma (MST), PTSD, and interpersonal violence (IPV), which can increase their risk of AID and/or pregnancy complications.^2,17^ WV are 1.6 times more likely to experience lifetime IPV than male Veterans.^18^ This is concerning as history of IPV increases the risk of suicidal ideation and self-harm behaviors in WV by twofold,^19^ as well as increased participation in high-risk pregnancy behaviors (e.g., smoking, alcohol, and drug use) and lower likelihood of seeking social support from a partner.^20^ More than 20% of WV screen positive for MST, compared with about 1% of male Veterans, with a positive screen associated with increased risk of PTSD, depression, anxiety, and substance use disorders.^17^ Pregnant Veterans with active PTSD were more likely to experience preeclampsia/eclampsia.^21^ The risk of developing an AID in military personnel with a history of PTSD was 58% higher compared with Veterans without a history of PTSD.^2^ In this same population, ≥6 antidepressant dispensations were associated with spontaneous preterm birth.^21^ The presence of trauma and subsequent mental health issues in WV influences their risk of AID and subsequent reproductive health outcomes and should be considered in their pregnancy care.

Despite the increased risks faced by WV of the development of AID and related critical threats to their reproductive health, relatively little is known about their reproductive outcomes. Thus, we sought to characterize pregnancy outcomes, AID disease outcomes, and past exposures to violence among women with AID.

## Methods

We conducted a case series study nested in a multisite cohort study. Pregnancy experiences and clinical outcomes were extracted from the Center for Maternal and Infant Outcomes Research in Translation (COMFORT).^22^ COMFORT is a longitudinal, multisite cohort study of pregnant and postpartum WV at 15 VA facilities, approved by the Veterans Administration Central Institutional Review Board.^22^ Veterans were invited to participate following pregnancy confirmation. Enrolled Veterans participated in two telephone surveys at 18–20 weeks gestation and within 3 months after delivery. Surveys explored pregnancy planning, contraceptive use, history of exposure to multiple forms of violence, pregnancy outcomes, and breastfeeding. Study data were collected and managed using REDCap, a secure, web-based application. Additionally, electronic medical record (EMR) data on participants who provided a social security number were accessed through the VA Corporate Data Warehouse.

To identify WV in the COMFORT cohort with an AID, EMRs were screened using diagnostic International Classification of Diseases 9 and 10 codes and for commonly used medications in AID (e.g., hydroxychloroquine, tumor necrosis factor inhibitors). To be included in this study, Veterans must have been evaluated by a subspecialist (rheumatologist, gastroenterologist, dermatologist, etc.) to confirm their AID. Veterans diagnosed with an AID after the registered pregnancy were excluded. A protocol for chart review and extraction was created by a rheumatologist and reviewed by another collaborating rheumatologist. Two pilot chart extractions from eligible patients were performed by two rheumatologists and a women’s health expert to ensure the accuracy of information. We attempted to collect serologies, imaging, disease characteristics (i.e., end organ damage), and subspecialty notes from outside the VA health care system; however, we found this information was inconsistently documented or unavailable.

Pregnancy and infant outcomes along with disease characteristics, including diagnosis, presence of autoantibodies, and end-organ damage, were collected by the piloted protocol. Pregnancy outcomes are synthesized in this study as a descriptive case series. Outcomes reported include history of trauma (MST, IPV, and PTSD), pregnancy complications, delivery type (vaginal vs. caesarean), gestational age, and newborn outcomes (birth weight, need for neonatal intensive care unit [NICU], and need for newborn hospitalization after delivery discharge). We also examined use of contraceptives prior to pregnancy to assess number of conceptions in Veterans with active contraceptive prescriptions. We report descriptive statistics (*n*, %; mean ± standard deviation [SD]) as appropriate. Self-reported race was combined into Black, White, or other race categories. Analysis was performed in SAS (SAS Institute Inc., Cary, NC., USA).

## Results

### Patient characteristics

Twenty-five WV with a mean age of 32 years (SD 4.92) were included in the analysis. White women comprised the majority of the sample (64%), while more than a third (36%) identified as Black or Other race and 20% reported Hispanic ethnicity. Included patients were diagnosed with more than 10 distinct types of AID, with the most frequently reported inflammatory bowel disease (*n* = 16%), psoriasis (*n* = 16%), and undifferentiated connective tissue disease (*n* = 16%) (Table 1). More than half of women (56%) reported a history of MST and nearly a third (28%) had been previously diagnosed with PTSD. In total, 17 women in this sample reported some form of exposure to violence (i.e., MST, PTSD, IPV). The mean number of past pregnancies was 2 (SD 1.46), with 36% of women reporting a prior pregnancy loss (Table 2).

**Table 1.** Women Veteran Demographics

Demographic (*n* = 25, unless otherwise noted)	Result
Age (years), mean (SD)	32.00 (4.92)
Race	
* Black*	6 (24%)
* Other*	3 (12%)
* White*	16 (64%)
Ethnicity	
* Hispanic*	5 (20%)
Autoimmune Condition	
* Inflammatory bowel disease*	4
* Psoriasis*	4
* Undifferentiated connective tissue disease*	4
* Spondyloarthropathies*	3
* Sjogren’s disease*	2
* Systemic lupus erythematosus (SLE)*	2
* Sarcoidosis*	2
* Rheumatoid arthritis (RA)*	1
* RA/SLE overlap*	1
* Vasculitis*	1
* Mixed connective tissue disease*	1
Number of children in the household (*n* = 24)	
* 0*	11 (46%)
* 1*	8 (33%)
* *≥ 2	5 (21%)
Prenatal Mental Health	
* Anxiety diagnosis (by a VA provider) during pregnancy*	2 (8%)
* Depression diagnosis (by a VA provider) during pregnancy*	3 (12%)
* Prenatal depression (Edinburgh Postnatal Depression Scale)*	
* *<8 (depression not likely)	19 (76%)
* *9–11 (depression possible)	0 (0%)
12–13 (fairly high possibility of depression)	2 (8%)
* *>14 (probably depression)	4 (16%)
History of Military Sexual Trauma	
* Uninvited and unwanted sexual attention (i.e., touching, cornering, pressure for sexual favors, or sexual remarks) during military service*	Yes: 14 (56%) No: 9 (36%) Declined: 2 (8%)
* Use of force or threat of force to have sexual contact against your will during military service*	Yes: 7 (28%) No: 16 (64%) Declined: 2 (8%)
History of PTSD	
* Ever diagnosed with PTSD?*	Yes: 7 (28%) No: 18 (72%)
History of Intimate Partner Violence	
* Has a current or former partner ever physically hurt you in the past 12 months? (n* = 16*)*	Never: 15 (94%) Rarely: 1 (6%)
* Has a current or former partner ever insulted you in the past 12 months? (n* = 15*)*	Never: 12 (80%) Rarely: 1 (7%) Sometimes: 2 (13%)
* Has a current or former partner ever threatened to harm you in the past 12 months? (n* = 16*)*	Never: 16 (100%)
* Has a current or former partner ever screamed or cursed at you in the past 12 months? (n* = 16*)*	Never: 14 (88%) Rarely: 1 (6%) Sometimes: 1 (6%)
* Has a current or former partner ever forced you to have sexual activities in the past 12 months? (n* = 16*)*	Never: 16 (100%)
Employment Status	
* Employed for wages*	12 (48%)
* Unemployed, looking for work*	1 (4%)
* Unemployed, not looking for work*	3 (12%)
* Homemaker*	5 (20%)
* Student*	3 (12%)
* Retired*	1 (4%)
Type of health insurance	
* Private*	Yes: 7 (28%) No: 17 (68%) Did not know: 1 (4%)
* Government*	Yes: 6 (24%) No: 19 (76%)

PTSD, post-traumatic stress disorder; SD, standard deviation.

**Table 2.** Outcomes of Prior Pregnancies

Pregnancy characteristic (*n* = 25)	Outcome
Number of past pregnancies, mean (SD)	2 (1.46)
Outcomes of prior pregnancies	
* Live birth*	14 (56%)
* Miscarriage*	9 (36%)
* Abortion*	2 (8%)
* Stillbirth*	1 (4%)

Twenty percent of Veterans were using contraception at the time of conception, including spermicide (foam or jelly), oral contraceptives, and a transdermal patch. The majority of women (92%) did not require the use of assisted reproductive technology for conception. The first prenatal visit most frequently occurred during the first trimester (84%), but some Veterans (16%) did not receive this care until 13 weeks gestation or later (Table 3).

**Table 3.** Characteristics of Current Pregnancy

Pregnancy characteristic (*n* = 25, unless otherwise noted)	Outcome
Conception	
Use of contraception at the time of conception	Yes: 5 (20%) *Foam or jelly (n* = 1) *Oral contraceptives (n* = 2) *Patch (n* = 1) No: 20 (80%)
Use of artificial reproductive technology	Yes: 2 (8%) No: 23 (92%)
Prenatal Care	
Weeks gestation at first prenatal visit	
* ≤8 weeks*	14 (56%)
* 9–12 weeks*	7 (28%)
* ≥13 weeks*	4 (16%)
Received prenatal care as early as desired by mother	Yes: 22 (88%) No: 3 (12%) *Difficulty obtaining VA* *paperwork needed to* *authorize for prenatal care* *(n* = 3)
Pregnancy Care	
Use of maternity care coordinator (MCC) services	Yes: 23 (92%) No: 2 (8%)
Frequency of contact with MCC (*n* = 23)	
* Less than once per month*	10 (44%)
* Once per month*	12 (52%)
* More than once per month*	1 (4%)
Type of insurance	
* VA maternity care benefits*	22 (88%)
* VA maternity care benefits and* * Private insurance*	3 (12%)
Appointments with VA providers during pregnancy	
* Yes, primary care*	8 (32%)
* Yes, mental health*	3 (12%)
* Yes, subspecialist*	8 (32%)
* No*	10 (40%)
Pregnancy Outcomes	
Gestational age at delivery (weeks), mean (SD)	37.73 (2.27)
Induced	13 (52%)
Type of delivery (*n* = 22)	
* Vaginal*	18 (82%)
* Cesarean*	4 (18%) *Planned: 3* *Unplanned: 1*
Pregnancy Complications	Yes: 11 (44%) No: 8 (32%) Missing: 6 (24%)
* Gestational diabetes*	1 (4%)
* Gestational hypertension*	2 (8%)
* Preeclampsia/eclampsia*	4 (16%)
* Preterm labor/delivery*	2 (8%)
Length of hospitalization, mother (*n* = 21)	
* ≤72 hours*	19 (90%)
* 4–7 days*	2 (10%)
Newborn weight (*n* = 22)	
* *≤5.8lbs	4 (18%)
* *5 lbs 9 ounces–8 lbs 16 ounces	18 (82%)
Need for neonatal intensive care unit (NICU) hospitalization (*n* = 20)	Yes: 4 (20%) *<4 days (1)* *≥1 week (3)* No: 16 (80%)
Length of hospitalization, newborn (*n* = 21)	
* ≤72 hours*	17 (81%)
* 4–7 days*	1 (5%)
* >7 days*	3 (14%)
Need for newborn hospitalization since postpartum discharge (*n* = 20)	Yes: 0 (0%) No: 20 (100%)
Overall newborn health (*n* = 21)	
* Excellent*	11 (52%)
* Very good*	9 (43%)
* Good*	1 (5%)
Postpartum Care	
Tried breastfeeding (*n* = 20)	Yes: 18 (90%) No: 2 (10%)
Postpartum depression (Edinburgh Postnatal Depression Scale) (*n* = 22)	
* ≤8 (depression not likely)*	17 (77%)
* 9–11 (depression possible)*	3 (13%)
* 12–13 (fairly high possibility of depression)*	1 (5%)
* ≥14 (probable depression)*	1 (5%)
Number of months to VA PCP follow-up (*n* = 22)	
* 1–3 months*	14 (63%)
* 4–6 months*	3 (14%)
* 7–9 months*	2 (9%)
* >9 months*	3 (14%)
Number of months to subspecialist follow-up (VA or community care) (*n* = 21)	
* 1–3 months*	12 (57%)
* 4–6 months*	3 (14%)
* 7–9 months*	1 (5%)
* >9 months*	5 (24%)
Seen in a VA emergency department before PCP or subspecialty follow-up	Yes: 5 (20%) No: 20 (80%)

PCP, Primary care physician.

Maternity care coordinator (MCC) services were used in most pregnancies with Veterans reporting (*n* = 23) contact with their MCC monthly (52%), less than monthly (44%), and more frequent than monthly (4%). Some Veterans (40%) did not seek health care at the VA during their pregnancy. Veterans reporting use of VA health care services during pregnancy (60%) were seen by their primary care provider (32%), subspecialist (32%), and/or mental health care provider (12%) (Table 3).

Of the Veterans with available pregnancy outcomes (*n* = 22), most experienced a vaginal delivery (82%) at full term (mean gestational age: 37.7 weeks). There were two nonlive births reported: one due to a blighted ovum in the first trimester and the second was a fetal demise at 34 weeks gestation. The blighted ovum was terminated with a medical abortion. The pregnancy with a fetal demise was terminated in a cesarean delivery due to patient preference. It is unclear if the mother consented to a fetal autopsy, and the cause of the fetal demise was undetermined. Three Veterans were lost to follow-up.

Forty-four percent of Veterans reported pregnancy complications, including preeclampsia/eclampsia (16%), preterm labor/delivery (8%), gestational diabetes (4%), and gestational hypertension (8%). Most mothers (90%) spent 3 days or less hospitalized after delivery (Table 3). Comorbid mental health conditions, including anxiety and depression, were diagnosed by a VA provider during pregnancy in 8% and 12% of WV, respectively (Table 1). Few newborns of WV in our sample met criteria for intrauterine growth restriction (IUGR) (<5 pounds, 8 ounces; 18%) or required NICU hospitalization (20%). Three newborns were hospitalized in the NICU for longer than 7 days. Following discharge, mothers (*n* = 21) rated their child’s health as excellent (52%), very good (43%), and good (5%) (Table 3).

More than half (57%) of Veterans were evaluated by the subspecialist managing their autoimmune condition within 3 months of delivery, and an additional three patients arranged follow-up within 6 months of delivery. However, 24% of Veterans were seen by their subspecialist more than 9 months after delivery. Twenty percent of Veterans were evaluated in a VA emergency department prior to their PCP or subspecialty follow-up for back pain, abdominal pain following a medical abortion in the pregnancy that was terminated, medication refills, and headache (Table 3).

## Discussion

This case series explores the outcomes of pregnant WV with AID, who receive care in the VA health care system in order to identify opportunities to improve medical care, continuity, and access to Veteran-specific resources for WV during pregnancy and in the postpartum period. We found that among the 25 identified Veterans, most utilized the services of an MCC, but many did not continue following with VA providers during pregnancy. Patients with AID often have multiple specialists to manage disease activity, therapies, and associated complications. Their engagement with patients during pregnancy is critical to optimize pregnancy outcomes. While most pregnancies resulted in live births, almost half of women in this study experienced pregnancy complications. It is possible Veterans received specialty care in the community during pregnancy, but the loss of their VA specialty provider contributes to segmented medical care and may impact integration back into the VA health care system in the postpartum.

In addition to the physical health needs associated with their AID, the identified cases had additional Veteran-specific risk factors that impact reproductive outcomes, including exposure to traumatic experiences and subsequent anxiety and depression. More than half of our participants reported experiencing MST, which may be explained by the increased risk of AID in Veterans with a history of violence.^2,23^ A meta-analysis, including military personnel and Veterans, demonstrated an estimated 38% of women have experienced MST.^24^ In WV with AID, the risk for concomitant depression and anxiety is higher due to the demands of their chronic and complex illness, in addition to the stressors of pregnancy and the postpartum period. For example, patients with psoriatic arthritis, spondyloarthropathies, and rheumatoid arthritis are more likely to experience postpartum depression compared to women without autoimmune disease (adjusted hazard ratio 1.22).^25^ Keeping WV with AID integrated in the VA health care system during pregnancy is important as mental health comorbidities are a Veteran-specific risk factor, which can be best addressed by providers and support staff with specialized training in these areas. This reinforces the importance of continuity of care and access to VA mental health services in our Veteran population, which should extend into peripartum and pregnancy care.

More than a third of Veterans in this case series reported a prior miscarriage, which is higher than the rates reported in the general population (13%) and women with inflammatory bowel disease (16%).^26,27^ This increased risk of adverse pregnancy outcomes among WV with AID could be attributed to disease activity, medication exposures, end-organ damage, and comorbidities.^28–31^ Due to their increased risk, it is critical that Veterans with AID experience planned pregnancies during periods of low disease activity to mitigate poor outcomes. However, in our sample, 20% of Veterans conceived while utilizing contraception. Notably, none of these Veterans were using a highly effective form of contraception (e.g., intrauterine device, subdermal implant), which may be more appropriate for this population.

Most Veterans in our sample successfully received pregnancy care during the first trimester. Our study’s observation of the first prenatal visit within the first 12 weeks was comparable with general population studies without AID (84% vs. 92%).^32^ The first trimester is a critical time in pregnancy due to organogenesis, which can be adversely impacted by AID activity and many AID medications, including teratogenic medications and medications with unknown safety profiles in pregnancy.^33^ The presence of specific autoantibodies, such as anti-Ro and antiphospholipid antibodies, can also impact the need for immunosuppressive medications and screening fetal ultrasounds starting in the first trimester.^33^

When a Veteran with an AID becomes pregnant, they can experience complications such as worsening of disease activity, need for medication changes, specialized imaging, and lab studies.^33,34^ An interdisciplinary approach among providers, obstetricians, and subspecialists ensures all aspects of the patient’s health care are addressed.^35^ Once a WV becomes pregnant, they are referred to a community provider enrolled in the VA Community Care Network for pregnancy care with a non-VA obstetrician.^36^ An MCC acts as a liaison between the VA and community to ensure Veterans have access to required resources and obstetric providers.^37^ Our study found that 40% of Veterans did not seek health care at the VA during pregnancy, and of those who did, only 32% were seen by a subspecialist. Subspecialty care is critical during pregnancy in this high-risk population. The American College of Rheumatology recommends evaluation of AID activity at least once per trimester.^33^ It is possible that WV are receiving subspecialist care during pregnancy from providers outside the VA without the knowledge of VA-based providers, resulting in fragmented care with prior VA specialists. Limited regional access to subspecialists, both within and outside the VA, who are comfortable managing pregnant patients and have specific training in women’s health care needs, is another challenge of receiving optimal peripartum care for WV with AID.

While the majority of Veterans in our sample experienced successful live births at full term, a concerning number experienced complications. Worldwide, 2%–8% of pregnancies are complicated by preeclampsia.^38^ In our study, 16% of women experienced preeclampsia/eclampsia, and 8% were diagnosed with gestational hypertension. Increased risk of preeclampsia has been previously reported in pregnant women with autoimmune conditions.^39^ For example, similar outcomes for preeclampsia (16.9%) have been observed in women with lupus nephritis.^39^ Our observed rate of pregnancy complications can likely be attributed to both disease activity related to underlying autoimmune conditions and systemic racism experienced by women from minoritized populations. To address the increased risk of preeclampsia associated with AID in pregnancy, obstetricians and subspecialists can consider more aggressive pregnancy care starting in the first trimester, including home blood pressure monitoring, frequent nursing visits, and consultation with maternal fetal medicine.

While WV with AID have increased risk of pregnancy complications and poor pregnancy outcomes, this case study demonstrated mostly live births with healthy newborns. Eighteen percent of newborns met criteria for IUGR (<5 pounds, 8 ounces), which is a described pregnancy complication in women with AID and mothers with history of trauma.^28,40^ However, the observed rate of IUGR in this study is lower than the general population (18% vs. 24%).^41^ Twenty percent of newborns required NICU care, which is comparable with the general population (10%–15%).^42^ Overall, most newborns were deemed in excellent or very good health by their mothers, and none required rehospitalization following discharge after delivery.

## Limitations

This case series has several limitations. First, we used a case series approach, which, while including a racially and ethnically diverse patient population, is descriptive in nature and broader conclusions cannot be determined about pregnancy outcomes in the greater WV population with AID. Our study likely missed Veterans with early pregnancy loss prior to enrollment in COMFORT, which may lead to the underrepresentation of first-trimester spontaneous abortions. Due to poor documentation and lack of access to non-VA clinic notes, disease characteristics could not be extracted, limiting our understanding of the extent of AID manifestations. Our results cannot be generalized to samples outside of Veterans with AID receiving health care through the VA.

## Conclusions

WV with AID have unique pregnancy risks for both the health of the mother and baby. Most Veterans in this case series did not receive care from their VA AID subspecialist during pregnancy. Future studies are needed to assess if pregnant Veterans receive subspecialty care for their AID outside the VA or if this management is provided by obstetrics during pregnancy, and how this impacts the continuity of their medical care and reintegration into the VA health care system in the postpartum period. Veteran-specific risk factors were observed, including the presence of prior traumatic experiences that can influence pregnancy outcomes in this high-risk population. This study provides an example of the complicated interface among the Veteran population, chronic and complex diseases, and reproductive outcomes that have long-term impacts on maternal and neonate health.

## Abbreviations Used

AID: Autoimmune disease
COMFORT: Center for Maternal and Infant Outcomes Research in Translation
EMR: Electronic medical record
IPV: Interpersonal violence
IUGR: Intrauterine growth restriction
MCC: Maternity care coordinator
MST: Military sexual trauma
NICU: Neonatal intensive care unit
PTSD: Post-traumatic stress disorder
PCP: Primary Care Physician
RA: Rheumatoid arthritis
SD: Standard deviation
SLE: Systemic lupus erythematosus
VA: Veterans Affairs
WV: Women Veterans
